# Increased albuminuria is highly prevalent in the general population: prevalence of CKD in the Gutenberg Health Study

**DOI:** 10.1093/ckj/sfaf399

**Published:** 2025-12-22

**Authors:** Daniel Kraus, Alexander Gieswinkel, Simone Cosima Boedecker-Lips, Pascal Klimpke, Marco Stortz, Eva M Schleicher, Jörn M Schattenberg, Norbert Pfeiffer, Jasmin Ghaemi, Irene Schmidtmann, Karl J Lackner, Oliver Tüscher, Thomas Münzel, Philipp S Wild, Peter R Galle, Julia Weinmann-Menke

**Affiliations:** I. Department of Medicine, University Medical Center of the Johannes Gutenberg University Mainz, Mainz, Germany; Preventive Cardiology and Preventive Medicine – Center for Cardiology, University Medical Center of the Johannes Gutenberg University Mainz, Mainz, Germany; I. Department of Medicine, University Medical Center of the Johannes Gutenberg University Mainz, Mainz, Germany; I. Department of Medicine, University Medical Center of the Johannes Gutenberg University Mainz, Mainz, Germany; I. Department of Medicine, University Medical Center of the Johannes Gutenberg University Mainz, Mainz, Germany; I. Department of Medicine, University Medical Center of the Johannes Gutenberg University Mainz, Mainz, Germany; Department of Internal Medicine II, Saarland University Medical Centre, Homburg, Germany; Department of Ophthalmology, University Medical Center of the Johannes Gutenberg University Mainz, Mainz, Germany; Department of Psychosomatic Medicine and Psychotherapy, University Medical Center of the Johannes Gutenberg University Mainz, Mainz, Germany; Institute for Medical Biometry, Epidemiology and Informatics, University Medical Center of the Johannes Gutenberg University Mainz, Mainz, Germany; Institute of Clinical Chemistry and Laboratory Medicine, University Medical Center of the Johannes Gutenberg University Mainz, Mainz, Germany; Department of Psychiatry and Psychotherapy, University Medical Center of the Johannes Gutenberg University Mainz, Mainz, Germany; Institute of Molecular Biology (IMB), Mainz, Germany; Department of Cardiology – Cardiology I, University Medical Center of the Johannes Gutenberg University Mainz, Mainz, Germany; Preventive Cardiology and Preventive Medicine – Center for Cardiology, University Medical Center of the Johannes Gutenberg University Mainz, Mainz, Germany; Institute of Molecular Biology (IMB), Mainz, Germany; Center for Thrombosis and Hemostasis (CTH), University Medical Center of the Johannes Gutenberg University Mainz, Mainz, Germany; German Center for Cardiovascular Research (DZHK), Partner Site Rhine-Main, Mainz, Germany; I. Department of Medicine, University Medical Center of the Johannes Gutenberg University Mainz, Mainz, Germany; I. Department of Medicine, University Medical Center of the Johannes Gutenberg University Mainz, Mainz, Germany; Center of Immunotherapy Mainz (FZI), Johannes Gutenberg University Mainz, Mainz, Germany

**Keywords:** chronic kidney disease, disease progression, increased albuminuria, population health, prevention

## Abstract

**Background:**

Early diagnosis of chronic kidney disease (CKD) is essential to slow progression and delay or prevent dialysis. However, in the absence of specific symptoms, patients and physicians may remain unaware of the disease for a long period. Here we present an analysis from the Gutenberg Health Study, a prospective longitudinal cohort study, to estimate the prevalence of CKD indicators in the population.

**Methods:**

A representative sample of 10 125 individuals underwent extensive medical testing at baseline; 9331 were tested again after 5 years. The estimated glomerular filtration rate (eGFR) was calculated using the Chronic Kidney Disease Epidemiology Collaboration formula with serum creatinine. Urinary albumin:creatinine ratios (UACRs) were determined from spot urine samples.

**Results:**

At baseline, 2.5% of subjects had decreased eGFR (<60 ml/min/1.73 m^2^), 10.4% had increased albuminuria (UACR >30 mg/g) and 1.0% had both. Within 5 years, the incidence of new-onset decreased eGFR was 3.4%, the incidence of new-onset increased albuminuria was 6.9% and 1.4% had both new-onset decreased eGFR and increased albuminuria. Most importantly, 6.8% of all subjects and 3.2% of subjects without hypertension, diabetes or known kidney disease had chronic increased albuminuria, consistent with the presence of CKD.

**Conclusions:**

This is the first study to report the longitudinal prevalence of CKD in the population. Chronic increased albuminuria, a sensitive marker of CKD, is highly prevalent in the German population even in the absence of risk factors for kidney disease.

## GRAPHICAL ABSTRACT

**GRAPHICAL ABSTRACT:**
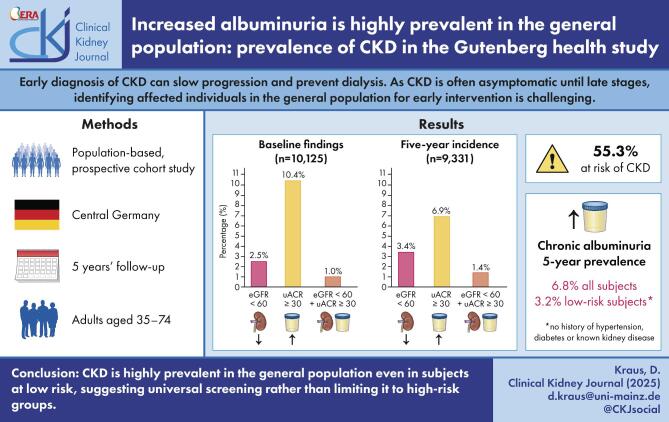

KEY LEARNING POINTS **What was known:**

- Chronic kidney disease (CKD) often goes unnoticed by patients and clinicians but may place a high burden on individuals and health systems as it advances.
- In the absence of systematic screening, little is known about the prevalence and incidence of CKD.
- Early detection of (asymptomatic) CKD may reduce morbidity and mortality and healthcare costs in the long term.

**This study adds:**

- This study assesses the prevalence and incidence of CKD in the population-based Gutenberg Health Study.
- Increased albuminuria, which is a sensitive indicator of kidney disease, is chronically present in 6.8% of the population.
- Knowledge of the prevalence of CKD in the population may inform decisions to implement screening programs.

**Potential impact:**

- With the advancement of novel therapies that delay kidney disease progression, early commencement of therapy may prevent morbidity and mortality, such as the need to perform dialysis or cardiovascular disease.

## INTRODUCTION

Chronic kidney disease (CKD), similar to arterial hypertension, type 2 diabetes mellitus and metabolic dysfunction–associated steatotic liver disease (MASLD), often develops unnoticed by patients and physicians. In the early stages, the diagnosis is frequently made by chance. In later stages, uraemia, hypervolaemia and other signs and symptoms may prompt investigations, but the damage will be irreversible at that point. To slow down disease progression and delay or prevent the need for kidney replacement therapy, early diagnosis is crucial [1].

Population screening may help detect subjects at risk of developing CKD. Estimates of the prevalence of CKD are needed to develop screening programs and assess their medical efficacy and cost-effectiveness.

Previously published population studies reported prevalences of decreased glomerular filtration rate (GFR) and/or increased albuminuria between 3.3% in Norway and 17.3% in Germany [2]. The Global Burden of Disease study assessed the prevalence of CKD in 2017 at 9.1% globally. However, all of these studies were based on single time points and, as such, could not validly detect the presence of CKD, which requires abnormalities of kidney structure and/or function to be present for at least 3 months according to the Kidney Disease: Improving Global Outcomes (KDIGO) definition [3].

GFR is routinely estimated from serum creatinine levels, which do not allow for early detection of slowly developing kidney disease. Furthermore, measuring creatinine requires drawing blood, and the prospect of needing a vein tap may represent an obstacle for people to participate in population screening. On the other hand, albuminuria is indeed an early marker of kidney damage and can be detected in a simple urine sample [4]. Recently, the THOMAS study (NCT04295889) demonstrated the feasibility of albuminuria screening in the Dutch population [5]. In addition, impairment in glomerular endothelial function likely plays a major role in the development of albuminuria and CKD progression. Glomerular endothelial dysfunction may reflect systemic microvascular dysfunction, accounting in part for the greater cardiovascular risk in patients with albuminuria [6].

The Gutenberg Health Study (GHS) is a large, ongoing, prospective population-based cohort study that follows a representative sample of ≈15 000 subjects from the city of Mainz and the surrounding county [7]. Mainz is situated in the Rhine-Main metropolitan area, a densely populated region in western Germany, home to many industries, academic institutions, regional governments and Germany’s largest airport. (The GHS is named after Johannes Gutenberg, a citizen of Mainz and inventor of the printing press.) Here we analyse GHS data from the baseline and the 5-year follow-up visits to estimate the prevalence and incidence of decreased GFR and/or albuminuria in the general population.

## MATERIALS AND METHODS

### Study population

The current study is an analysis of the GHS, which selected a representative sample of 15 010 subjects ages 35–74 years from the citizen registries of the city of Mainz and the surrounding county (county Mainz-Bingen). The initial sample was randomly selected from the local registry offices in the city and the county. The sample was stratified for sex, age decade and urban versus rural residency. The age range was chosen because the primary aim of the GHS when it was conceived was to study cardiovascular outcomes [7]. Recruitment began in April 2007 with a comprehensive medical workup; the last baseline visit took place in March 2012. The current analysis includes data from the baseline visits and the first follow-up visit, to which subjects were invited after 5 years. Details of the study protocol have been published elsewhere [7]. The GHS adheres to the principles of the Declaration of Helsinki and was approved by the Ethics Commission of the State Chamber of Physicians of Rhineland-Palatinate (reference no. 837.020.07). All included subjects provided informed written consent.

### Diagnoses

The GHS records the diagnoses of arterial hypertension, diabetes and CKD. Arterial hypertension is considered present if the visit systolic blood pressure (BP) is >140 mmHg, if the visit diastolic BP is >90 mmHg or if the participant takes antihypertensive medication; self-disclosed diagnoses of hypertension are not included in this definition. Diabetes is considered present if the glycosylated haemoglobin (HbA1c) level is ≥6.5% or if the participant takes antiglycaemic medication; self-disclosed diagnoses of diabetes are not included in this definition. CKD is considered present if the participant reports has been told he/she has kidney disease and if this was treated within the past 12 months. Obesity was defined as a body mass index (BMI; body weight divided by the squared height in meters) of ≥30 kg/m^2^. In addition, all participants are asked to self-identify as having been diagnosed with hypertension, diabetes or CKD.

### Biochemical analyses

**Figure 1:**
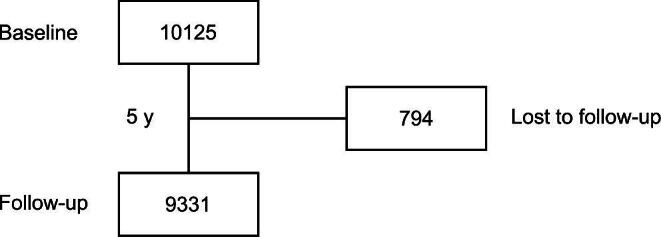
CONSORT diagram. CONSORT flow diagram of the study population at baseline and after 5 years.

Serum and urine creatinine were measured by a modified Jaffe method with an Architect c8000 or c16000 instrument (Abbott Diagnostics, Wiesbaden, Germany), traceable to NIST SRM 967. Estimated GFR was calculated using the 2009 Chronic Kidney Disease Epidemiology Collaboration equation [8]. Urinary albumin was measured in random spot urine by an immunonephelometric assay (BN Prospec, Siemens Healthineers, Eschborn, Germany). The urinary albumin:creatinine ratio (UACR) in the spot urine was used to categorize albuminuria according to the KDIGO: A1, UACR <30 mg/g creatinine; A2, UACR 30–300 mg/g creatinine; and A3, UACR >300 mg/g creatinine [3]. A total of 7333 subjects (48.9%) had albuminuria below the lower limit of detection (LLD; 12 mg/l) with creatininuria above the LLD (40 mg/dl); 224 subjects (1.5%) had albuminuria above the LLD but creatininuria below the LLD; and 4013 subjects (26.7%) had both albuminuria below the LLD and creatininuria below the LLD. Clinically meaningful UACRs were calculated for 10 125 subjects with urinary albumin concentrations (numerator) above the LLD and/or urinary creatinine levels (denominator) above the LLD (Fig. 1).

### Statistical analysis

Continuous variables are described by mean values and standard deviation (SD) or median and interquartile range (IQR) if they are skewed (|skewness| >1). Discrete variables are described through relative and absolute frequencies. *P*-values are estimated via *t*-test for mean (SD), Mann–Whitney U-test for median (IQR) and chi-squared test for dichotomous variables. Confidence intervals (CIs) for proportions were calculated using an equality of proportions test. An alpha level of 0.05 is used as the threshold for statistical significance. All analyses were carried out with R version 4.2.1 (R Foundation for Statistical Computing, Vienna, Austria) [9].

## RESULTS

### Baseline

At baseline, 44.2% of participants reported having hypertension; a diagnosis of hypertension (as per the GHS definition) was made in 50.9% (Table 1). A total of 8.0% of participants reported having diabetes and 10.8% met the GHS definition; 15.3% of participants reported being diagnosed with kidney disease, but only 1.2% had been treated for kidney disease in the past 12 months (Table 1); and 28.1% were obese (Table 1).

**Table 1:** Baseline characteristics.

Characteristics	All	Women	Men	*P* for sex
Biometric information				
Patients, *n* (%)	10 125 (100)	4011 (39.6)	6114 (60.4)	<.0001
Age (years), mean (SD)	55.1 (11.2)	54.8 (11.4)	55.3 (11.1)	.0057
Height (m), mean (SD)	1.72 (0.09)	1.64 (0.07)	1.77 (0.07)	<.0001
Weight (kg), mean (SD)	82.3 (16.8)	73.6 (16.0)	88.0 (14.7)	<.0001
BMI (kg/m^2^), median (IQR)	27.1 (23.9–30.2)	26.3 (23.1–30.7)	27.5 (25.1–30.4)	<.0001
Systolic BP (mmHg), mean (SD)	131.5 (17.3)	128.1 (18.3)	133.7 (16.2)	<.0001
Diastolic BP (mmHg), mean (SD)	82.5 (9.6)	80.7 (9.5)	83.6 (9.5)	<.0001
Heart rate (bpm), mean (SD)	69.1 (11.0)	70.5 (10.6)	68.2 (11.1)	<.0001
Smoking status, *n*/*N* (%)				
Smoking (ever)	5618/10 105 (55.6)	1872/4002 (46.8)	3746/6103 (61.4)	<.0001
Smoking (occasionally)	136/10 105 (1.3)	46/4002 (1.1)	90/6103 (1.5)	NS
Smoking (daily)	1919/10 105 (19.0)	721/4002 (18.0)	1198/6103 (19.6)	<.05
Cardiovascular disease history				
Previous myocardial infarction	332/10 073 (3.3)	274/6075 (4.5)	58/3998 (1.5)	<.0001
Previous stroke	212/10 072 (2.1)	143/6075 (2.4)	69/3997 (1.7)	.033
Congestive heart failure	356/10 125 (3.5)	182/4939 (3.7)	174/2991 (5.8)	<.0001
Diagnoses (GHS definition), *n*/*N* (%)				
Hypertension	5156/10 119 (51.0)	1793/4007 (44.7)	3363/6112 (55.0)	<.0001
Diabetes	1089/10 108 (10.8)	350/3995 (8.8)	739/6113 (12.1)	<.0001
Obesity	2848/10 122 (28.1)	1140/4010 (28.3)	1708/6112 (27.9)	.60
Kidney disease	117/10 119 (1.2)	44/4009 (1.1)	73/6110 (1.2)	.70
Patient-reported medical history, *n*/*N* (%)				
Hypertension	4439/10 045 (44.2)	1593/3983 (40.0)	2846/6062 (46.9)	<.0001
Diabetes	807/10 047 (8.0)	253/3976 (6.4)	554/6071 (9.1)	<.0001
Kidney disease	1541/10 055 (15.3)	770/3985 (19.3)	771/6070 (12.7)	<.0001
Proteinuria	61/9952 (0.6)	25/3946 (0.6)	36/6006 (0.6)	.49
Medication, *n*/*N* (%)				
ACEi	1531/10 011 (15.3)	458/3976 (11.5)	1073/6035 (17.8)	<.0001
ARB	1071/10 011 (10.7)	388/3976 (9.8)	683/6035 (11.3)	.014
MRA	49/10 011 (0.5)	11/3976 (0.3)	38/6035 (0.6)	.013
SGLT2i^a^	47/10 011 (0.5)	11/3976 (0.3)	36/6035 (0.6)	.024

All participants identified themselves as either woman or man.

ACEi: angiotensin-converting enzyme inhibitor; ARB: angiotensin receptor blocker; bpm: beats per minute; MRA: mineralocorticoid receptor antagonist; NS: not significant.

^a^SGLT2i were initially approved in Germany in 2012, i.e. at the end of the baseline visits period of the GHS (2007–2012).

A total of 26.5% of participants were treated with an angiotensin-converting enzyme inhibitor, an angiotensin receptor blocker or a mineralocorticoid receptor antagonist (Table 1). Only 0.5% were treated with a sodium–glucose co-transporter 2 inhibitor (SGLT2i); however, the first SGLT2i was approved in Germany in the last year of the baseline visit period (2012).

### Population at risk of developing kidney disease

At baseline, 5400 participants (53.3%) had any combination of hypertension, diabetes or CKD; these were considered at-risk for progressive kidney disease (Table 2); 4725 (46.7%) had no diagnosis and were considered low risk (Table 2). Low-risk participants were younger, more likely to be male and, remarkably, more often daily smokers (Table 2). (Smoking status is not included in the definition of being at risk for kidney disease for the purpose of this analysis.) 

**Table 2:** Baseline characteristics according to risk group.

Characteristics	No CKD, DM, or HTN	Any of CKD, DM, HTN	CKD	HTN	DM
Patients, *n*/*N* (%)	4725/10 125 (46.7)	5400/10 125 (53.3)	117/10 125 (1.2)	5156/10 125 (50.9)	1089/10 125 (10.8)
Biometric information					
Women, *n*/*N* (%)	2123/4725 (44.9)	1888/5400 (35.0)	44/117 (37.6)	1793/5156 (34.8)	350/1089 (32.1)
Age (years), mean (SD)	50.2 (10.5)	59.4 (10.0)	58.9 (10.1)	59.5 (10.0)	62.6 (8.6)
Height (m), mean (SD)	1.72 (0.09)	1.71 (0.09)	1.70 (0.09)	1.71 (0.09)	1.70 (0.09)
Weight (kg), mean (SD)	77.6 (15.2)	86.4 (17.1)	84.0 (15.9)	86.5 (17.0)	91.7 (18.5)
BMI (kg/m^2^), median (IQR)	25.5 (23.1–28.3)	28.7 (25.9–32.1)	28.6 (26.2–31.4)	28.7 (25.9–32.2)	30.9 (27.4–34.6)
Smoking status, *n*/*N* (%)					
Smoking (ever)	2598/4718 (55.1)	3020/5387 (56.1)	85/157 (57.8)	2869/5145 (55.8)	666/1085 (61.4)
Smoking (occasionally)	87/4718 (1.8)	49/5387 (0.9)	1/116 (0.9)	46/5145 (0.9)	10/1085 (0.9)
Smoking (daily)	1082/4718 (22.9)	837/5387 (15.5)	21/116 (18.1)	778/5145 (15.1)	171/1085 (15.8)
Patient-reported medical history, *n*/*N* (%)					
Kidney disease	584/4690 (12.5)	957/5365 (17.8)	117/117 (100.0)	888/5122 (17.3)	201/1083 (18.6)
Hypertension	464/4691 (9.9)	3975/5354 (74.2)	72/117 (61.5)	3925/5115 (76.7)	832/1078 (77.2)
Diabetes	0/4704 (0)	807/5343 (15.1)	15/117 (12.8)	677/5101 (13.3)	807/1070 (75.4)
Medication, *n*/*N* (%)					
ACEi	78/4651 (1.7)	1453/5360 (7.1)	29/117 (24.8)	1420/5117 (27.8)	410/1087 (37.7)
ARB	32/4651 (0.7)	1039/5360 (19.4)	27/117 (23.1)	1034/5117 (20.2)	276/1087 (25.4)
MRA	4/4651 (0.1)	45/5360 (0.8)	4/117 (3.4)	42/5117 (0.8)	24/1087 (2.2)
SGLT2i^a^	0/4651 (0)	47/5360 (0.9)	0/117 (0)	41/5117 (0.8)	47/1087 (4.3)

Risk groups are subset according to the GHS definitions for CKD, hypertension and diabetes. Continuous variables are described by mean values and SD or with median (IQR) if they are skew (|Skewness| >1). Discrete variables are described through relative and absolute frequencies.

ACEi: angiotensin-converting enzyme inhibitor; ARB: angiotensin receptor blocker; MRA: mineralocorticoid receptor antagonist.

^a^SGLT2i were initially approved in Germany in 2012; baseline visits took place between 2007 and 2012.

Of note, 12.5% of those in the no-CKD, no-hypertension, no-diabetes group reported having kidney disease, but were not adjudicated as having CKD, because they did not report having had their kidney disease treated in the past 12 months (Table 2).

Of 117 participants whose kidney disease had been treated in the past 12 months, 62 (53%) had hypertension, 4 (3.4%) had diabetes and 14 (12%) had both hypertension and diabetes. Importantly, 37 (31.6%) of the 117 participants with treated CKD had neither hypertension nor diabetes diagnosed.

### Prevalence of indicators of CKD

**Figure 2:**
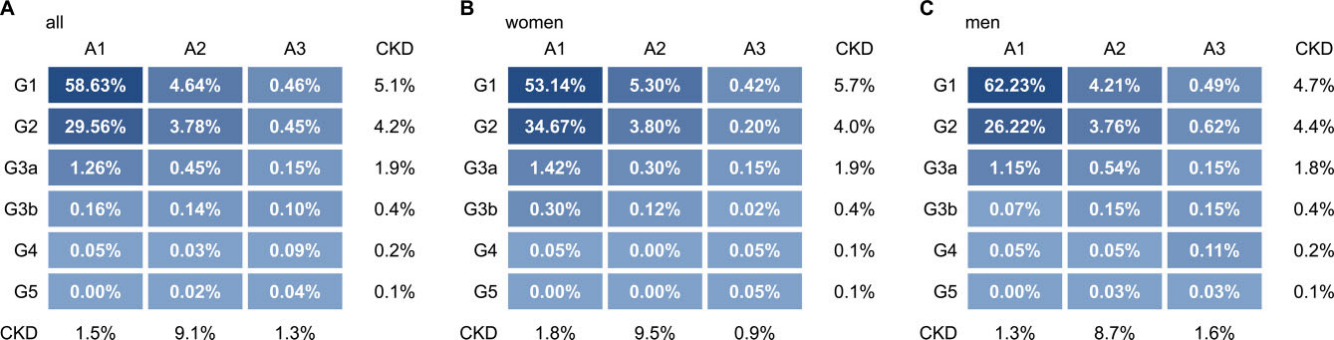
KDIGO risk categories. Proportion of subjects in the eGFR and UACR risk categories defined by the KDIGO: **(A)** all subjects, **(B)** women and **(C)** men. A1, UACR <30 mg/g; A2, UACR 30–300 mg/g; A3, UACR >300 mg/g; the rows and columns labelled ‘CKD’ contain the sums of percentages in the categories corresponding to CKD categories (G1–G2 A2–A3 and G3a–G5 A1–A3). G1, eGFR ≥90 ml/min/1.73 m^2^; G2, eGFR 60–89 ml/min/1.73 m^2^; G3a, eGFR 45–59 ml/min/1.73 m^2^; G3b, eGFR 30–44 ml/min/1.73 m^2^; G4, eGFR 15–29 ml/min/1.73 m^2^; G5, eGFR <15 ml/min/1.73 m^2^.

The KDIGO ‘heat map’ describes the risk of progressive kidney disease according to six categories of decreasing eGFR (G1, G2, G3a, G3b, G4, G5) and three categories of increasing albuminuria (A1, A2, A3) [3]. At baseline, 88.2% of the GHS cohort could be classified as G1 A1 or G2 A1, consistent with the absence of CKD (disregarding possible pathological diagnoses in a few cases) (Fig. 2). The remaining 11.8% had eGFRs <60 ml/min/1.73 m^2^ (category G3a and higher) or albuminuria with UACRs >30 mg/g (categories A2 and A3), or both (Fig. 2). Importantly, the majority of these had increased albuminuria but not decreased eGFR (Fig. 2).

**Figure 3:**
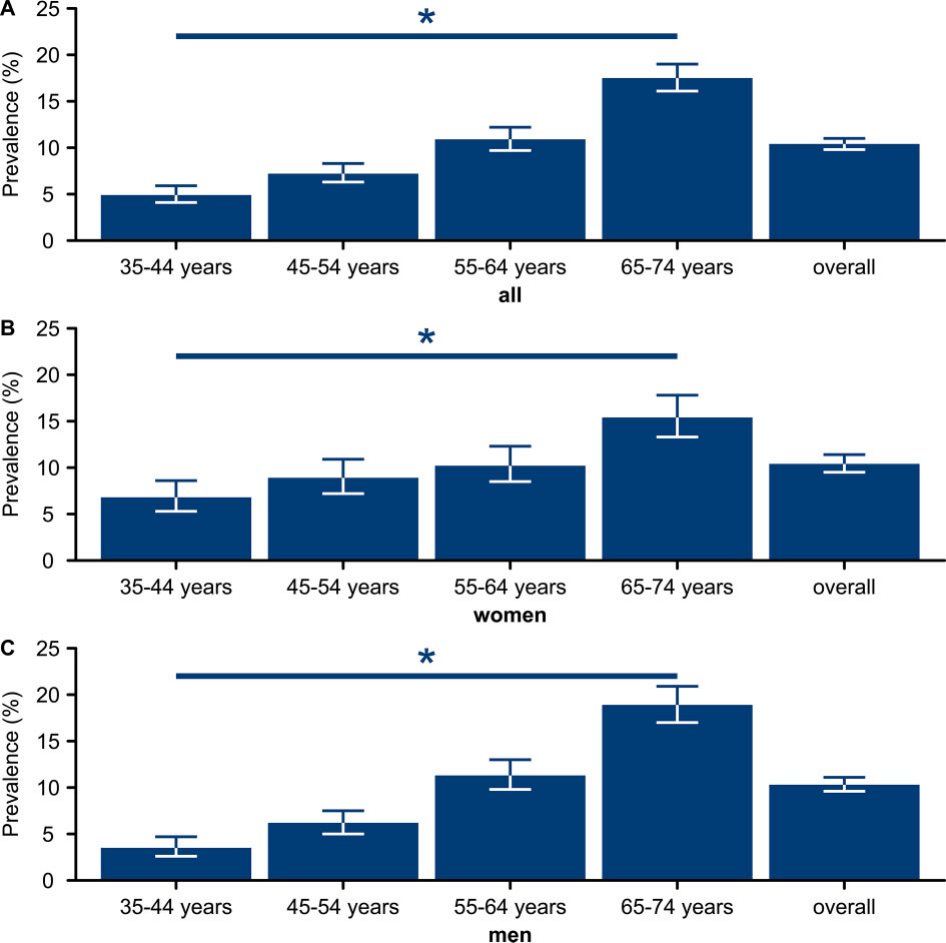
Albuminuria by age group. Prevalence of albuminuria (spot UACR ≥30 mg/g) stratified by age: **(A)** all subjects, **(B)** women and **(C)** men. Whiskers indicate 95% CIs. *P < .05 by linear regression.

The point prevalences of decreased eGFR (<60 ml/min/1.73 m^2^) and of increased albuminuria at baseline both increased with age, and this trend was statistically significant (Fig. 3 and Table 3). In the oldest age group (65–74 years), the combination of reduced eGFR and increased albuminuria was present in 2.6% of patients (Table 3). Increased albuminuria with uACR >300 mg/g and reduced eGFR (<60 ml/min/1.73 m^2^) in combination with uACR >30 mg/g was more prevalent in men than in women (Table 4).

**Table 3:** Prevalence of indicators of kidney disease by age group.

Characteristics	Overall	35–44 years	45–54 years	55–64 years	65–74 years
eGFR categories					
<60 ml/min/1.73 m^2^	2.5 (2.2–2.8)	0.2 (0.1–0.5)	0.4 (0.2–0.8)	2.0 (1.6–2.7)	6.8 (5.9–7.9)
<30 ml/min/1.73 m^2^	0.2 (0.1–0.3)	0.1 (0.0–0.3)	0.1 (0.0–0.4)	0.1 (0.0–0.4)	0.6 (0.4–1.0)]
UACR categories					
≥30 mg/g	10.4 (9.8–11.0)	4.9 (4.1–5.9)	7.2 (6.3–8.3)	10.9 (9.7–12.2)	17.5 (16.1–19.0)
≥300 mg/g	1.3 (1.1–1.5)	0.6 (0.3–1.0)	0.7 (0.5–1.2)	1.2 (0.8–1.7)	2.5 (2.0–3.2)
UACR categories with eGFR <60 ml/min/1.73 m^2^					
≥30 mg/g	1.0 (0.8–1.2)	0.1 (0.0–0.4)	0.3 (0.1–0.6)	0.8 (0.5–1.2)	2.6 (2.1–3.3)
≥300 mg/g	0.4 (0.3–0.5)	0.0 (0.0–0.3)	0.2 (0.0–0.4)	0.3 (0.1–0.6)	1.0 (0.7–1.5)
UACR categories with eGFR <30 ml/min/1.73 m^2^					
≥30 mg/g	0.2 (0.1–0.3)	0.0 (0.0–0.3)	0.1 (0.0–0.4)	0.1 (0.0–0.3)	0.5 (0.2–0.8)
≥300 mg/g	0.1 (0.1–0.2)	0.0 (0.0–0.3)	0.1 (0.0–0.4)	0 (0–0.2)	0.3 (0.2–0.7)

Values are presented as percentages (95% CIs) as determined by the chi-squared test for trend in proportions.

**Table 4:** Prevalence of indicators of kidney disease by sex.

Characteristics	Women	Men	*P*-value
eGFR categories			
<60 ml/min/1.73 m^2^	2.5 (2.0–3.0)	2.5 (2.1–2.9)	1.00
<30 ml/min/1.73 m^2^	0.1 (0.1–0.3)	0.3 (0.2–0.5)	0.21
UACR categories			
≥ 30 mg/g	10.4 (9.5–11.4)	10.3 (9.6–11.1)	0.87
≥ 300 mg/g	0.9 (0.6–1.3)	1.6 (1.3–1.9)	0.0039
UACR categories with eGFR <60 ml/min/1.73 m^2^			
≥30 mg/g	0.7 (0.5–1.0)	1.2 (1.0–1.5)	0.014
≥300 mg/g	0.3 (0.1–0.5)	0.4 (0.3–0.7)	0.24
UACR categories with eGFR <30 ml/min/1.73 m^2^			
≥30 mg/g	0.1 (0.0–0.3)	0.2 (0.1–0.4)	0.15
≥300 mg/g	0.1 (0.0–0.3)	0.1 (0.1–0.3)	0.58

Values are presented as percentages (95% CIs) as determined by the chi-squared test for trend in proportions.

Weighting the GHS data for the population in Germany in 2021, the prevalence of eGFR categories G3a and higher was 2.2% (women, 2.3%; men, 2.1%) and the prevalence of UACR categories A2 and higher was 9.9% (women, 10.3%; men, 9.6%) (Table 5).

**Table 5:** Prevalence of indicators of kidney disease weighted for the 2021 German population.

Characteristics	All	Women	Men
eGFR categories			
<60 ml/min/1.73 m^2^	2.2 (1.9–2.5)	2.3 (1.9–2.9)	2.1 (1.8–2.5)
<30 ml/min/1.73 m^2^	0.2 (0.1–0.3)	0.1 (0.1–0.3)	0.2 (0.1–0.4)
UACR categories with any eGFR			
≥30 mg/g	9.9 (9.3–10.5)	10.3 (9.4–11.3)	9.6 (8.9–10.4)
≥300 mg/g	1.2 (1.0–1.4)	0.9 (0.6–1.2)	1.4 (1.1–1.8)
UACR categories with eGFR <60 ml/min/1.73 m^2^			
≥30 mg/g	0.9 (0.7–1.1)	0.7 (0.4–1.0)	1.1 (0.8–1.4)
≥300 mg/g	0.3 (0.2–0.5)	0.3 (0.1–0.5)	0.4 (0.2–0.6)
UACR categories with eGFR <30 ml/min/1.73 m^2^			
≥30 mg/g	0.2 (0.1–0.3)	0.1 (0.0–0.3)	0.2 (0.1–0.4)
≥300 mg/g	0.1 (0.1–0.2)	0.1 (0.0–0.3)	0.1 (0.1–0.3)

Numbers indicate percentages for the 2021 German population determined by inverse probability weighting of the GHS sample. Numbers in parentheses indicate 95% CIs determined by the chi-squared test for trend in proportions.

Even in low-risk subjects, i.e. those who do not have a history of hypertension, diabetes or CKD, the point prevalence of increased albuminuria was 5.5% (Table 6). Prevalence was higher in women than in men and increased across age groups (Table 6).

**Table 6:** Prevalence of indicators of CKD without known CKD risk.

				Age (years)			
CKD indicator	All	Women	Men	35–44	45–54	55–64	65–74
eGFR <60 ml/min/1.73 m^2^	0.3 (0.2–0.6)	0.4 (0.2–0.8)	0.3 (0.2–0.6)	0 (0–0.3)	0 (0–0.3)	0.7 (0.3–1.5)	1.2 (0.6–2.4)
UACR >30 mg/g	5.5 (4.8–6.2)	7.1 (6.0–8.3)	4.3 (3.6–5.1)	3.7 (2.9–4.8)	5.0 (4.0–6.3)	5.9 (4.6–7.6)	9.4 (7.4–11.8)
eGFR <60 ml/min/1.73 m^2^ and/or UACR >30 mg/g	5.7 (5.1–6.4)	7.4 (6.3–8.7)	4.5 (3.8–5.3)	3.7 (2.9–4.8)	5.0 (4.0–6.3)	6.5 (5.1–8.3)	10.2 (8.2–12.7)

Only subjects who did not report having hypertension, diabetes or kidney disease.

Numbers in parentheses indicate 95% CIs determined by the chi-squared test for trend in proportions.

### Incidence of indicators of CKD

**Figure 4:**
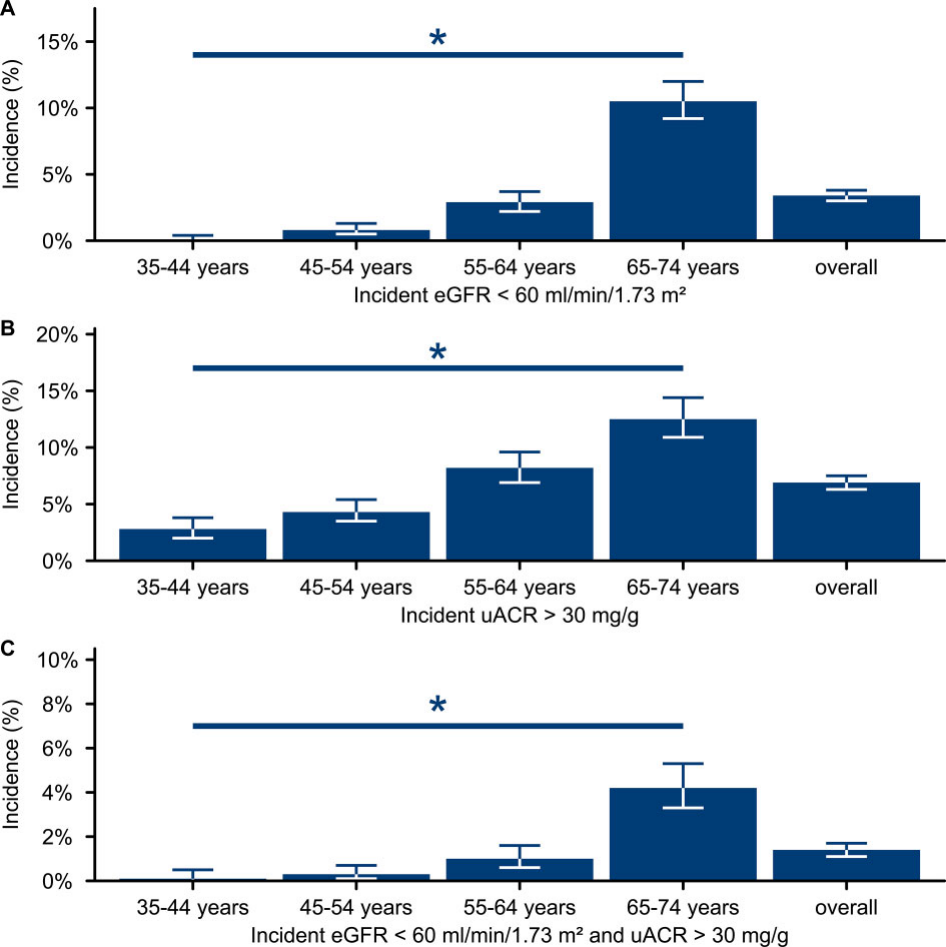
Incidence of indicators of CKD. Incidence of indicators of CKD between the baseline visit and 5-year follow-up. **(A)** Incident eGFR <60 ml/min/1.73 m^2^, **(B)** incident increased albuminuria and **(C)** incident eGFR <60 ml/min/1.73 m^2^ with incident albuminuria. Whiskers indicate 95% CIs. *P < .05 by linear regression.

Of the 9331 subjects who presented to the first GHS follow-up visit after 5 years and had UACR measurements, 3.4% had an incident eGFR <60 ml/min/1.73 m^2^, 6.9% had an incident UACR >30 mg/g and 1.4% had an incident eGFR <60 ml/min/1.73 m^2^ as well as incident UACR >30 mg/g (Fig. 4, Table 7). The incidence was age-dependent: subjects between 35 and 44 years of age had an incident eGFR <60 ml/min/1.73 m^2^ in 0.1% of cases, but among participants ages 65–74 years, 10.5% had an incident eGFR <60 ml/min/1.73 m^2^; 4.2% additionally developed increased albuminuria (Fig. 4, Table 7).

**Table 7:** Incidence of indicators of kidney disease by age group and sex.

Indicator	Overall	35–44 years	45–54 years	55–64 years	65–74 years
Incident eGFR <60 ml/min/1.73 m^2^					
Women	3.4 (2.8–4.2)	0.1 (0.0–0.8)	0.8 (0.4–1.8)	2.6 (1.7–4.1)	11.2 (9.0–13.8)
Men	3.4 (2.9–3.9)	0.1 (0.0–0.6)	0.8 (0.4–1.5)	3.0 (2.2–4.1)	10.1 (8.4–12.0)
Incident UACR >30 mg/g					
Women	6.7 (5.7–7.9)	4.1 (2.6–6.2)	4.0 (2.6–6.0)	7.8 (5.8–10.5)	11.5 (8.9–14.7)
Men	6.9 (6.2–7.8)	2.1 (1.3–3.3)	4.5 (3.4–5.9)	8.3 (6.8–10.1)	13.1 (11.0–15.5)
Incident eGFR <60 ml/min/1.73 m^2^ and UACR >30 mg/g					
Women	1.0 (0.7–1.5)	0.2 (0.0–1.1)	0.3 (0.1–1.3)	0.2 (0.0–1.0)	3.5 (2.2–5.4)
Men	1.6 (1.3–2.0%)	0.1 (0.0–0.7)	0.2 (0.1–0.8)	1.4 (0.9–2.3)	4.6 (3.5–6.1)

Numbers in parentheses indicate 95% CIs determined by the chi-squared test for trend in proportions.

Reasons for not appearing for the follow-up visit were participants’ decision not to attend (65.1%), no response (17.3%), death (13.8%) and exclusion (3.9%).

### Prevalence of chronic increased albuminuria

**Figure 5:**
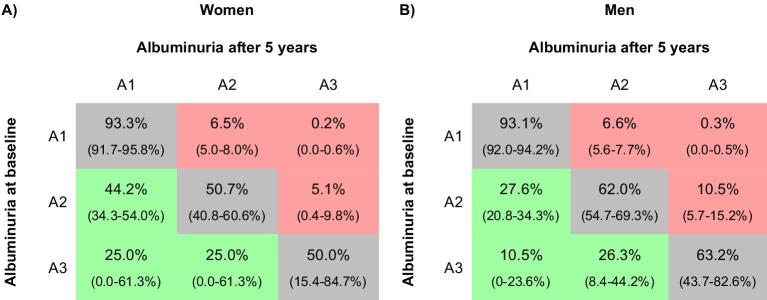
Change in KDIGO albuminuria category over time (all subjects). The percentage of all subjects in each albuminuria category after 5 years compared with the category at baseline: **(A)** women and **(B)** men. 95% CIs are given in parentheses. KDIGO albuminuria risk categories: A1, UACR <30 mg/g; A2, UACR 30–300 mg/g; A3, UACR >300 mg/g.

An important criterion of CKD is persistence of structural damage, which can manifest as confirmed, chronic increased albuminuria. After 5 years, 109 women (50.7%) and 225 men (62.0%) who had had category A2 albuminuria at baseline remained in this category (Fig. 5); 95 women (44.2%) and 100 men (27.6%) with A2 at baseline were reclassified as A1 at follow-up. Of those presenting with A3 albuminuria at baseline, 8 women (50.0%) and 36 men (63.2%) remained in this category at follow-up (Fig. 5; for an extrapolation to the European standard population, see Supplementary Fig. 1). 

Importantly, regarding the whole group of subjects who returned for the follow-up visit, 6.8% had chronic increased albuminuria that persisted after 5 years.

**Figure 6:**
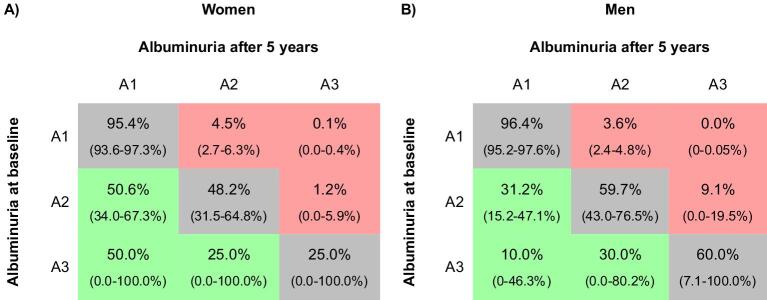
Change in KDIGO albuminuria category over time (no known hypertension, diabetes or CKD). The percentage of subjects that did not report having hypertension, diabetes or CKD in each albuminuria category after 5 years compared with the category at baseline: **(A)** women and **(B)** men. 95% CIs are given in parentheses. KDIGO albuminuria risk categories: A1, UACR <30 mg/g; A2, UACR 30–300 mg/g; A3, UACR >300 mg/g.

When only those subjects were considered who did not report having hypertension, diabetes or CKD, 39 women (48.2%) and 46 men (59.7%) who presented with A2 albuminuria at baseline also had A2 albuminuria at follow-up (Fig. 6; for an extrapolation to the European standard population, see Supplementary Fig. 2).

Regarding the whole group of low-risk subjects who returned for the follow-up visit, 3.2% had chronic increased albuminuria that persisted after 5 years.

## DISCUSSION

In the absence of typical signs and symptoms, CKD can only be diagnosed at early stages by chance or systematic screening. Some countries have implemented programs to screen for CKD in patients with diabetes, who are at high risk of developing diabetic kidney disease. However, CKD may develop entirely unnoticed in people without known chronic disease.

Estimates of the prevalence and incidence of CKD may aid in developing screening and prevention programs for the population at large. Here we report the prevalences and incidences of two important indicators of CKD, increased albuminuria and eGFR [10, 11], in a representative prospective, longitudinal cohort study of adults 35–74 years of age in Germany.

A notable finding of this analysis is the high point prevalence and incidence of increased albuminuria across all age groups: ≈1 in 10 participants had category A2 or A3 albuminuria at baseline and 5% of participants developed albuminuria (UACR >30 mg/g) within 5 years. The prevalence of increased albuminuria increased with age. The overall prevalence of impaired kidney function (eGFR <60 ml/min/1.73 m^2^; categories G3a and higher) was 2.5%; this was more strongly age dependent than increased albuminuria. After 5 years, ≈4% of participants in the oldest age group presented with newly developed impaired kidney function and increased albuminuria.

These results are in line with previous reports of point prevalences. For example, in the representative, cross-sectional DEGS1 study [12], which used semi-quantitative test strips to determine albuminuria, 11.5% of all subjects had increased albuminuria [13]. The north German SHIP-1 and south German KORA F4 studies reported point prevalences of increased albuminuria of 20.2% and 8.8%, respectively [14]. The earlier MONICA study, which examined non-diabetic adults from southern Germany in a cross-sectional manner, reported increased albuminuria in 8.0% of men and 7.5% of women [15]. A recent analysis of the Hamburg City Health Study found that 11% of the population had either UACR ≥30 mg/day and/or eGFR <60 ml/min/1.73 m^2^; 6% had increased albuminuria and decreased eGFR [16].

The high point prevalences of increased albuminuria may give rise to concerns about biological variability and possible methodological artefacts, even though the GHS employs immunological urinary albumin assays as opposed to dipstick assays. Indeed, a single measurement at baseline may overestimate the prevalence of chronic increased albuminuria, which, by definition, must persist for >3 months [17]. For instance, urinary tract infections, menses and exercise may result in false positive results, as well as posture, which comes into play if urine sampling occurs at random times, as in this study. Therefore, KDIGO mandates the confirmation of increased albuminuria even when the measurement itself is precise [17]. Despite these caveats, increased albuminuria is generally better suited for detecting structural damage to the kidneys early than eGFR computed from serum creatinine measurements, which are not particularly sensitive to incipient kidney disease.

Importantly, one of the strengths of the current study is its longitudinal design, which addresses most of these concerns. After 5 years, the increased albuminuria detected at the baseline visit was confirmed in >50% of women and 60% of men. Among the entire cohort, 6.8% of subjects had persistently increased albuminuria, and even among subjects without hypertension, diabetes or known kidney disease, the longitudinal prevalence of increased albuminuria was 3.2%. To our knowledge, this is the first population-based study to report chronic rather than point prevalences of CKD. It should be noted that these numbers likely underestimate the true prevalence of structural kidney disease. Within 5 years, subjects may have had changes in their lifestyle or medical management that reduce albuminuria, while the underlying structural glomerular damage persists.

As reported previously, the eGFR slope in the GHS cohort is approximately −1 ml/min/1.73 m^2^/year [18]. The current analysis translates into an overall incidence of reduced eGFR (<60 ml/min/1.73 m^2^) of 2.9% in 5 years. The incidence is low in younger adults but rises sharply at >55 years of age. Extrapolated to the German standard population, the overall prevalence of decreased eGFR of 2.2% agrees very well with the prevalence of 2.3% derived from the DEGS1 study across a wider age span (18–79 years) [13].

This analysis of the GHS is, to our knowledge, the first study that reports longitudinal prevalences of markers of CKD. Additional strengths of this study include its prospective and longitudinal design, which allows for the detection of true CKD, the use of precise quantitative albuminuria measurements rather than dipsticks and the calculation of eGFR from serum creatinine, a universally available and affordable biomarker. Furthermore, participation rates at baseline as well as at the 5-year follow-up visit were high. Because the results of the current analysis were not yet communicated to the participants, it is unlikely that lifestyle changes or improved medical management had a diminishing effect on the 5-year prevalence.

Limitations of this study include the random timing of urine sampling [19], the absence of urinalyses that could detect possible urinary tract infections and possible glomerulonephritis, the lack of measurements of novel biomarkers for kidney disease [20] and the calculation of eGFR from serum creatinine, which is not overly sensitive to changes in kidney function (but reflects the most widespread method to assess eGFR). An additional limitation of the study is the lower limit of detection of the urine assays. UACR could not be calculated for those subjects with both urinary albumin and urinary creatinine concentrations below the LLDs.

The cost-effectiveness of screening the general population for CKD strongly depends not only on the cost of screening but also on the availability, cost and efficacy of possible interventions and the cost of comorbidities that are associated with CKD, including cardiovascular disease. Until recently, these were limited to lifestyle modifications, which are notoriously hard to implement and maintain [21, 22], and drugs that block the renin–angiotensin system. Accordingly, screening the general population for CKD was not considered cost-effective [23, 24]. However, the armamentarium of drugs that retard CKD progression has grown considerably over the past few years. It will continue to do so, including novel drugs for specific aetiologies of glomerulonephritis. Interestingly, contemporary reports suggest that general screening for albuminuria may be cost-effective [5, 25], and interventions triggered by the detection of albuminuria may reduce the overall cardiovascular disease burden [26]. The amount of CKD-related healthcare spending correlates with disease progression [27, 28], and it is hoped that early diagnosis and retardation of progression not only saves people from having to face dialysis but also reduces the overall cost to the healthcare system [29]. Of note, the council of the European Renal Association has recommended screening for increased albuminuria for the general population [1], and it is also suggested as part of a systematic workup by the European Society of Cardiology [30], since increased albuminuria reflects endothelial dysfunction, an important biomarker linked to increased oxidative stress and inflammation [31] and an adverse cardiovascular prognosis. Whether screening may be cost-effective in a given population requires knowledge of the prevalence and overall health of this population.

Albuminuria is not only a marker of CKD but is a cardiovascular risk factor in its own right, even in the absence of overt CKD. In fact, increased albuminuria may serve as a biomarker for cardiovascular–kidney–metabolic disease [32]. Early detection of increased albuminuria may inform disease and risk management at an early stage, something that patient advocates have been calling for [10].

Too few participants of the GHS with indicators of CKD were aware of their disease. This is reminiscent of two other common diseases, hypertension and diabetes, which are also often underdiagnosed. At the same time, proportionally more subjects with hypertension or diabetes than subjects with CKD know their diagnosis, highlighting a need to improve detection of CKD even among subjects who are not at risk of kidney disease.

Importantly, while the frequency of CKD diagnoses in German health insurance claims data rose from 4.4% to 7.3% between 2013 and 2022 [33], only 64.8% of patients with a recorded diagnosis of CKD category G4 were actually referred to a nephrologist, and 85% of those subjects who were not referred to a specialist did not get a proteinuria workup [34].

In the current study, we show that CKD is highly prevalent in the GHS, even among low-risk subjects. Further investigations of the feasibility of general population screening and therapeutic interventions are warranted, with the aim of reducing morbidity and mortality that arise from undiagnosed CKD.

## Supplementary Material

sfaf399_Supplemental_File

## Data Availability

The analysis presents clinical data of a large-scale population-based cohort with ongoing follow-up examinations. This project constitutes a major scientific effort with high methodological standards and detailed guidelines for analysis and publication to ensure scientific analyses on highest level. Therefore, data are not made available for the scientific community outside the established and controlled workflows and algorithms. To meet the general idea of verification and reproducibility of scientific findings, we offer access to data in the local database in accordance with the ethics vote upon request at any time. The GHS steering committee, which comprises a member of each department involved and the coordinating principal investigator of the GHS (P.S.W.), convenes once a month. The steering committee decides on internal and external access of researchers and use of the data and biomaterials based on a research proposal supplied by the researcher. Interested researchers make their requests to the coordinating principal investigator of the GHS [P.S.W. (philipp.wild@unimedizin-mainz.de)]. More detailed contact information is available at the home page of the GHS (www.gutenberghealthstudy.org).
