# Supplementary material for: Increased albuminuria is highly prevalent in the general population: prevalence of CKD in the Gutenberg Health Study
Source: Clin Kidney J. 2025 Dec 22;19(2):sfaf399. doi: 10.1093/ckj/sfaf399 (PMC13014362; doi:10.1093/ckj/sfaf399)
Supplement: sfaf399_Supplemental_File [file sfaf399_Supplemental_File.docx]

Albuminuria is highly prevalent in the general population: Indicators of chronic kidney disease in the gutenberg health study

Daniel Kraus, Alexander Gieswinkel, Simone Cosima Boedecker-Lips, Pascal Klimpke, Marco Stortz, Eva M. Schleicher, Jörn M. Schattenberg, Norbert Pfeiffer, Jasmin Ghaemi, Irene Schmidtmann, Karl J. Lackner, Oliver Tüscher, Thomas Münzel, Philipp S. Wild, GHS Research Consortium, Peter R. Galle, Julia Weinmann-Menke

**Supplementary data**

# Figures

## Supplementary Figure 1: Change in KDIGO albuminuria category over time (all subjects, 1976 European standard population)

| A) |  | Women | | | B) |  | Men | | |
| --- | --- | --- | --- | --- | --- | --- | --- | --- | --- |
|  |  | Albuminuria after 5 years | | |  |  | Albuminuria after 5 years | | |
|  |  | A1 | A2 | A3 |  |  | A1 | A2 | A3 |
| Albuminuria at baseline | A1 | 94.1%  (92.6-95.5%) | 5.7%  (4.3-7.1%) | 0.2%  (0.0-0.6%) | Albuminuria at baseline | A1 | 94.3%  (93.3-95.3%) | 5.5%  (4.5-6.5%) | 0.2%  (0.0-0.4%) |
|  | A2 | 46.3%  (36.1-56.5%) | 49.3%  (39.0-59.5%) | 4.4%  (0.0-8.9%) |  | A2 | 30.6%  (22.9-38.3%) | 59.5%  (51.3-67.6%) | 10.0%  (4.9-15.1%) |
|  | A3 | 35.7%  (0.0-78.4%) | 21.4%  (0.0-59.0%) | 42.3%  (0-86.7%) |  | A3 | 8.7%  (0.0-22.4%) | 26.1%  (6.0-46.2%) | 65.2%  (43.6-86.9%) |

The figure shows the percentage of the computed European standard population in each albuminuria category after five years, compared to the category at baseline. **(A)** women; **(B)** men. 95% confidence intervals are given in parentheses. A1-A3: KDIGO albuminuria risk categories. A1, ACR < 30 mg/g; A2, ACR 30-300 mg/g; A3, ACR > 300 mg/g; ACR, albumin-creatinine ratio.

## Supplementary Figure 2: Change in KDIGO albuminuria category over time (no known hypertension, diabetes, or CKD; 1976 European standard population)

| A) |  | Women | | | B) |  | Men | | |
| --- | --- | --- | --- | --- | --- | --- | --- | --- | --- |
|  |  | Albuminuria after 5 years | | |  |  | Albuminuria after 5 years | | |
|  |  | A1 | A2 | A3 |  |  | A1 | A2 | A3 |
| Albuminuria at baseline | A1 | 95.6%  (93.9-97.3%) | 4.4%  (2.7-6.0%) | 0.1%  (0.0-0.4%) | Albuminuria at baseline | A1 | 96.8%  (95.7-97.9%) | 3.2%  (2.1-4.3%) | 0.0%  (0.0-0.1%) |
|  | A2 | 54.2%  (37.9-70.6%) | 44.6%  (28.3-60.9%) | 1.2%  (0.0-5.7%) |  | A2 | 33.3%  (16.2-50.5%) | 58.0%  (40.1-75.9%) | 8.7%  (0.0-19.6%) |
|  | A3 | 50.0%  (0.0-100.0%) | 25.0%  (0.0-100.0%) | 25.0%  (0.0-100.0%) |  | A3 | 20.0%  (0.0-65.1%) | 20.0%  (0.0-65.1%) | 60.0%  (7.1-100.0%) |

The figure shows the percentage of the computed European standard population that did not report having hypertension, diabetes, or CKD, in each albuminuria category after five years, compared to the category at baseline. 95% confidence intervals are given in parentheses. **(A)** women; **(B)** men. A1-A3: KDIGO albuminuria risk categories. A1, ACR < 30 mg/g; A2, ACR 30-300 mg/g; A3, ACR > 300 mg/g; ACR, albumin-creatinine ratio.

## Supplementary Table 1: Prevalence of increased albuminuria in high-risk groups not on antiproteinuric therapy at baseline

| Subgroup | Not on antiproteinuric therapy | Increased albuminuria |
| --- | --- | --- |
| Hypertension | 36.3 [35.2; 37.4] | 75.7 [72.9; 78.2] |
| Diabetes mellitus | 5.5 [5.0; 6.1] | 27.3 [24.6; 30.1] |
| Chronic kidney disease | 0.9 [0.7; 1.1] | 3.3 [2.4; 4.7] |

Subgroups refer to subjects with self-reported diagnoses. Antiproteinuric therapy is defined as any combination of angiotensin-converting enzyme inhibitor (ACEi), angiotensin receptor blocker (ARB), mineralocorticoid receptor antagonist (MRA), or sodium-glucose transporter-2 inhibitor (SGLT2i) therapy. Increased albuminuria is defined as urinary albumin-creatinine ratio (uACR) equal to or greater than 30 mg/g; the values in this column reflect the percentage of subjects with increased albuminuria who are not treated with antiproteinuric drugs in the respective subgroups.

## Supplementary Table 2: Incidence of indicators of CKD (standard populations)

|  | 2021 German standard population | 1976 European standard population |
| --- | --- | --- |
| Incident eGFR < 60 ml/min/1.73 m² | | |
| Women | 3.2% (2.7-3.9%) | 2.4% (1.9-3.0%) |
| Men | 2.9% (2.4-3.4%) | 2.3% (1.9-2.7%) |
| Incident ACR > 30 mg/g | | |
| Women | 6.6% (5.6-7.8%) | 5.9% (5.0-7.0%) |
| Men | 6.5% (5.7-7.3%) | 5.7% (5.0-6.5%) |
| Incident eGFR < 60 ml/min/1.73 m² and ACR > 30 mg/g | | |
| Women | 0.9% (0.6-1.4%) | 0.7% (0.4-1.1%) |
| Men | 1.4% (1.1-1.8%) | 1.1% (0.8-1.5%) |
